# Influence of risk factors on the long-term survival of oral rehabilitation with extra-narrow implants: a retrospective study

**DOI:** 10.1590/1678-7757-2022-0089

**Published:** 2022-08-01

**Authors:** Elcio MARCANTONIO, Ivete Aparecida de Mattias SARTORI, Camila Pereira VIANNA, Roberta Schroder ROCHA, Waleska CALDAS, Larissa Carvalho TROJAN

**Affiliations:** 1 Faculdade Ilapeo Curitiba Brasil Faculdade Ilapeo, Curitiba, Brasil.; 2 Neodent Curitiba Brasil Neodent, Curitiba, Brasil.; 3 Universidade Federal do Paraná Curitiba Brasil Universidade Federal do Paraná, Curitiba, Brasil.; 4 Straumann Group Andover USA Straumann Group, LLC, Andover, USA.

**Keywords:** Prostheses and implants, Survival rate, Risk factors, Smokers

## Abstract

**Objective:**

This study aimed to retrospectively collect clinical data to evaluate the influence of possible risk factors on the long-term success of implant treatment with extra-narrow (2.9 mm diameter) implants in a daily dental practice setting.

**Methodology:**

Data were collected from records of patients who received at least one extra-narrow implant from 2012 to 2017, regarding implant survival, prosthesis survival, patient characteristics, and implant characteristics. The association between the dependent variables “implant survival”, “prosthesis survival,” and “adverse events” related to patient and implant characteristics was statistically evaluated by chi-square tests. Moreover, implant and prosthesis survival were analyzed by Kaplan-Meier survival curves.

**Results:**

The sample was constituted of 58 patients (37 women and 21 men) with a mean age of 54.8 years old (SD: 12.5), followed up for up to eight years. In total, 86 extra-narrow implants were placed within this sample. Four implants were lost, resulting in an implant survival rate of 95.3%. A total of 55 prostheses were inserted and only one (1.8%) was lost, resulting in a prosthesis survival rate of 98.2%. The mean implant and prosthesis survival time was, respectively, 7.1 years and 6.3 years, according to the Kaplan-Meier survival analysis. A correlation was found between smoking and implant loss, which makes implant loss eight times more likely to occur in smokers than non-smokers. A significant association was also found between prosthesis loss and previous need of prosthesis repair. However, it was not considered clinically relevant. No association was found between the occurrence of adverse events and later implant or prosthesis loss.

**Conclusion:**

High implant and prosthesis survival rates were found in the long term for treatment with extra-narrow implants. Moreover, a significant correlation between smoking and implant loss was observed.

## Introduction

Dental implants are widely used with great success and long-term survival rates in completely and partially edentulous patients.^1,2^ The implant diameter choice is based on several factors and, since an adequate bone volume and interdental space are required to produce good results, single-tooth rehabilitation in the anterior region can be challenging.^3^ Moreover, when placed in the atrophic alveolar bone, standard-diameter implants can expose their threads and lead to failure.^4^This is common in cases of agenesis, present in 2.2% to 7.6% of the population,^5^ and after tooth extraction, in which the alveolar bone resorption is progressive.^4^ Other conditions, such as trauma, neoplasia, and denture wearing, are related to reduced space.^6^

Some treatment approaches are suggested to successfully manage patients with limited space for standard dental implants, such as bone augmentation techniques. However, these approaches are more invasive, presenting higher risks of complications, besides a longer time and additional costs.^7^ In cases of limited mesiodistal space, orthodontic treatment and adhesive partial denture are suggested, but it might not meet all patients’ expectations. Thus, narrow-diameter implants emerge as a reliable alternative.

Although there is no consensus in the literature on the definition of narrow implants, in general, implants with a diameter narrower than 3.5 mm are considered narrow whereas implants with diameters narrower than 3.0 mm are described as extra-narrow or mini implants.^8^ Reduced bleeding, postoperative discomfort, and healing time are some of the reported advantages of these implants when compared with grafting procedures.^4^ Moreover, narrow and extra-narrow implant survival rates from 80% to 100% were reported in a follow-up period of up to seven years.^5,6,9,10^

Regarding aesthetic aspects, which are especially important in the rehabilitation in the anterior region, good results seem to be produced by narrow implants.^11^ The reduced diameter makes it possible to achieve an adequate 3-dimensional position, respecting the necessary distance between implant and adjacent teeth, as well as surrounding bone, to facilitate papillae formation and its maintenance in the long term.^9^ This is especially important to achieve good aesthetic outcomes in upper and lower lateral incisors and central incisors, which present the smallest mesiodistal dimensions.^12^ However, possible mechanical complications and other risk factors must be considered since the reduced bone–implant contact may lead to implant fractures.^3^

Therefore, this study aimed to retrospectively collect clinical data to evaluate the influence of possible risk factors on the long-term survival of oral rehabilitation with extra-narrow (2.9 mm diameter) implants, in a daily dental practice setting.

## Methodology

### Sample and study parameters

This study was reviewed and approved by the local Research Ethics Committee (Araraquara, Brazil; approval no. 3.553.077). Inclusion criteria were patients who had at least one 2.9-mm-diameter implant (Facility, Neodent, Curitiba, Brazil), inserted at ILAPEO College (Curitiba, Brazil) from 2012 to 2017, whose records presented postoperative clinical follow-up data.

Data was retrospectively collected from the patients’ records, according to the following parameters:

Implant survival: implant survival was defined as no implant loss at each follow-up visit.

Prosthesis survival: prosthesis survival was defined as the prosthesis remaining *in situ* at each follow-up visit.

Risk factors: patient demographic and general health data; general data for implants and prosthesis abutments, type of loading, and adverse events occurred after surgery.

### Statistical analysis

All analyses were performed using SPSS 16.0 for Windows (SPSS Inc. Headquarters, Chicago, USA). Descriptive summary statistics were estimated for all parameters. Quantitative parameters were described by mean, standard deviation, median, quartiles, minimum, and maximum. For qualitative variables, frequencies were given. Survival rates were estimated by dividing the number of events by the total number of implants/prostheses evaluated.

The association between the dependent variables “implant survival,” “prosthesis survival,” and “adverse events” and patient and implant characteristics was evaluated by chi-square tests and by estimating the relative frequencies, odds ratios (OR), and 95% confidence intervals. Missing data concerning a specific parameter was not included in association analyses. It was not possible to estimate the odds ratio of several variables since there was not sufficient sample to perform the test.

Implant and prosthesis survival were further analyzed by Kaplan-Meier survival curves. Some factors could not be analyzed by Kaplan-Meier survival curves due to insufficient sample. The significance level for all tests was oblique p<0.05.

## Results

All patients rehabilitated with at least one extra-narrow implant from 2012 to 2017 at ILAPEO College were included. The sample was constituted of 58 patients, of which 37 (63.8%) were women and 21 (36.2%) men, with a mean age of 54.8 years old (SD: 2.5; range: from 23.7 to 83.9). A total of 86 extra-narrow (2.9 mm diameter) implants were placed. Their length ranged from 10 to 14 mm, to support single or multi-unit fixed and removable prostheses in maxilla and mandible. Patients were followed up for a mean period of 2.8 years (SD: 1.9; up to 8.0). Four implants were lost due to lack of osseointegration, resulting in an implant survival rate of 95.3%. Three of these losses occurred before loading.

The most frequent patients’ medical condition was psychological limitations (10;17.2%), followed by smoking habit (5; 8.6%), thyroid dysfunction (4; 6.9%), coagulation disorders (5.2%), bone metabolism disorders (3; 5.2%), severe bruxism (2; 3.4%), bisphosphonate therapy for more than one year (2; 3.4%), poor healing capacity (1; 1.7%), regular steroid use (1; 1.7%), and previous radiotherapy in the head/neck (1; 1.7%).

The correlation between patient-related variables and implant loss are shown in Table 1. A correlation was found between smoking and implant loss, which makes implant loss eight (95% CI 1.0–63.9) times more likely to occur in smokers than non-smokers (p=0.024).

**Table 1:** Relative frequencies of patient-related variables and their association with implant loss

Implant loss?		Yes		No		Total	OR (95% CI)	p-value
		N	%	N	%	N		
Sex	Woman	4	6.6	57	93.4%	61	ƚ	0.246
	Man	0	0.0	25	100.0%	25	(ref.)	
Presence of thyroid dysfunction?	Yes	1	25.0	3	75.0%	4	8.8 (0.7–111.3)	0.176
	No	3	3.7	79	96.3%	82	(ref.)	
Presence of coagulation disorders?	Yes	0	0.0	3	100.0%	3	ƚ	0.894
	No	3	3.8	77	96.2%	80	(ref.)	
Presence of poor healing capacity?	Yes	0	0.0	1	100.0%	1	ƚ	0.964
	No	3	3.7	79	96.3%	82	(ref.)	
Regular steroid use?	Yes	0	0.0	1	100.0%	1	ƚ	0.953
	No	4	4.8	80	95.2%	84	(ref.)	
Previous radiotherapy in the head/neck?	Yes	0	0.0	1	100.0%	1	ƚ	0.953
	No	4	4.8	80	95.2%	84	(ref.)	
Bisphosphonate therapy?	Yes	0	0.0	3	100.0%	3	ƚ	0.864
	No	4	4.9	78	95.1%	82	(ref.)	
Psychological limitations?	Yes	0	0.0	17	100.0%	17	ƚ	0.402
	No	4	5.9	64	94.1%	68	(ref.)	
Presence of bone metabolism disorders?	Yes	0	0.0	4	100.0%	4	ƚ	0.822
	No	4	4.9	77	95.1%	81	(ref.)	
Smoking?	Yes	2	18.2	9	81.8%	11	8.0 (1.0–63.9)	0.024*
	No	2	2.7	72	97.3%	74	(ref.)	

*Statistically significant at p<0.05.

ƚ No sufficient sample size to calculate.

Regarding implant-related variables, extra-narrow implants with 14 mm length were the most used ones (39; 45.3%). The most frequent insertion site was the lower central incisor (24; 27.9%), followed by the upper lateral incisor (19; 22.1%). Insertion torques from 36 to 60 N.cm were reached in most implants (39;45.3%). A peri-implant bone loss greater than 1.5 mm was observed in six (7.0%) implants. No significant association was found between implant characteristics and implant loss (Table 2).

**Table 2:** Relative frequencies of implant-related variables and their association with implant loss

Implant loss?		Yes		No		Total	OR (95% CI)	p-value
		N	%	N	%	N		
Implant length	10 mm	0	0.0%	17	100.0%	17	ƚ	
	12 mm	3	7.7%	36	92.3%	39	2.5 (0.2–22.8)	0.857
	14 mm	1	3.6%	27	96.4%	28	(ref)	
Region of implant placement	Lower canines	0	0.0%	4	100.0%	4	ƚ	
	Lower central incisors	1	2.9%	34	97.1%	35	0.2 (0.0–3.2)	0.745
	Lower molars	0	0.0%	3	100.0%	3	ƚ	
	Lower premolars	0	0.0%	7	100.0%	7	ƚ	
	Upper canines	0	0.0%	1	100.0%	1	ƚ	
	Upper lateral incisors	2	10.5%	17	89.5%	19	0.7 (0.0–9.3)	0.794
	Upper premolars	1	14.3%	6	85.7%	7	(ref)	
	Others	0	0.0%	10	100.0%	10	ƚ	
Insertion torque (N.cm)	10–35	1	4.5%	21	95.5%	22	0.6 (0.0–5.8)	0.636
	36–60	3	7.7%	36	92.3%	39	(ref)	
Prosthesis type	Multi-unit fixed prosthesis	0	0.0%	42	100.0%	42	ƚ	ƚ
	Overdenture	0	0.0%	13	100.0%	13	ƚ	
	Single-unit prosthesis	1	4.0%	24	96.0%	25	(ref)	
Time until loading	Immediate loading	0	0.0%	40	100.0%	40	ƚ	
	1–4 months	1	33.3%	2	66.7%	3	0.5 (0.0–8.9)	0.638
	5–12 months	0	0.0%	26	100.0%	26	ƚ	
	> 12 months	0	0.0%	11	100.0%	11	ƚ	
	Not loaded	3	50.0%	3	50.0%	6	(ref)	
Final prosthesis retention	Cemented	0	0.0%	15	100.0%	15	ƚ	ƚ
	Overdenture	0	0.0%	12	100.0%	12	ƚ	
	Screwed	0	0.0%	19	100.0%	19	(ref)	
Peri-implant bone loss?	Yes, more than 1.5 mm	1	16.7%	5	83.3%	62	3.5 (0.3–40.6)	0.311
	No	3	5.4%	53	94.6%	56	(ref)	
Any prosthesis repair?	Yes	0	0.0%	2	100.0%	2	ƚ	ƚ
	No	0	0.0%	48	100.0%	48	(ref)	

*Statistically significant at p<0.05.

Influence of risk factors on the long-term survival of oral rehabilitation with extra-narrow implants: a retrospective study

In total, 55 prostheses were inserted. Only one (1.8%) was lost, resulting in a prosthesis survival rate of 98.2%. Regarding the type of loading, 40 (46.5%) implants were immediately loaded whereas the other 40 were loaded after one month or more. Most implants were used in restorations supported by one extra-narrow implant (44; 51.2%). A total of 42 (48.8%) implants were used as support for multi-unit fixed prostheses, 25 (29.1%) for single-unit prostheses, and 13 (15.1%) for overdentures. For the other six implants, the prothesis type was not reported.

No correlation was found between patients’ medical conditions and prosthesis loss (Table 3). However, a significant association was found between prosthesis loss and previous need of prosthesis repair (p=0.040; Table 4).

**Table 3:** Relative frequency of patient-related variables and their association with prosthesis loss

Prosthesis loss?		Yes		No		Total		p-value
		N	%	N	%	N	%	
Sex	Woman	0	0.0%	30	100.0%	30	100.0%	0.072
	Man	1	5.0%	19	95.0%	20	100.0%	
Presence of thyroid dysfunction?	Yes	0	0.0%	2	100.0%	2	100.0%	0.824
	No	1	2.1%	47	97.9%	48	100.0%	
Presence of coagulation disorders?	Yes	0	0.0%	2	100.0%	2	100.0%	0.861
	No	1	2.2%	45	97.8%	46	100.0%	
Presence of poor healing capacity?	Yes	0	0.0%	0	0.0%	0	0.0%	0.952
	No	1	2.1%	47	97.9%	48	100.0%	
Presence of incomplete jawbone growth?	Yes	0	0.0%	0	0.0%	0	0.0%	0.783
	No	1	2.0%	49	98.0%	50	100.0%	
Regular steroid use?	Yes	0	0.0%	0	0.0%	0	0.0%	0.953
	No	1	2.0%	48	98.0%	49	100.0%	
Previous radiotherapy in the head/neck?	Yes	0	0.0%	1	100.0%	1	100.0%	0.953
	No	1	2.1%	47	97.9%	48	100.0%	
Bisphosphonate therapy?	Yes	0	0.0%	1	100.0%	1	100.0%	0.864
	No	1	2.1%	47	97.9%	48	100.0%	
Psychological limitations?	Yes	0	0.0%	8	100.0%	8	100.0%	0.402
	No	1	2.4%	40	97.6%	41	100.0%	
Presence of bone metabolism disorders?	Yes	0	0.0%	2	100.0%	2	100.0%	0.822
	No	1	2.1%	46	97.9%	47	100.0%	
Smoking?	Yes	0	0.0%	3	100.0%	3	100.0%	0.568
	No	1	2.2%	45	97.8%	46	100.0%	

**Table 4:** Relative frequency of implant-related variables and their association with prosthesis loss

Prosthesis loss?		Yes		No		Total		p-value
		**N**	**%**	**N**	**%**	**N**	**%**	
Implant length	10 mm	0	0.0%	11	100.0%	11	100.0%	0.383
	12 mm	1	5.9%	16	94.1%	17	100.0%	
	14 mm	0	0.0%	21	100.0%	21	100.0%	
	15 mm	0	0.0%	0	0.0%	0	0.0%	
Region of implant placement	Lower canines	0	0.0%	1	100.0%	1	100.0%	0.972
	Lower central incisors	1	4.5%	21	95.5%	22	100.0%	
	Lower molars	0	0.0%	2	100.0%	2	100.0%	
	Lower premolars	0	0.0%	3	100.0%	3	100.0%	
	Upper canines	0	0.0%	0	0.0%	0	0.0%	
	Upper lateral incisors	0	0.0%	9	100.0%	9	100.0%	
	Upper premolars	0	0.0%	4	100.0%	4	100.0%	
	Others	0	0.0%	9	100.0%	9	100.0%	
Insertion torque (N.cm)	10–35	0	0.0%	15	100.0%	15	100.0%	0.605
	36–60	1	4.3%	22	95.7%	23	100.0%	
Healing	Yes	0	0.0%	24	100.0%	24	100.0%	0.520
	No	1	3.8%	25	96.2%	26	100.0%	
Prosthesis type	Multi-unit fixed prosthesis	0	0.0%	22	100.0%	22	100.0%	0.338
	Overdenture	0	0.0%	12	100.0%	12	100.0%	
	Single-unit prosthesis	1	6.2%	15	93.8%	16	100.0%	
Time between implant and prosthesis placement	Immediate loading	1	3.0%	32	97.0%	33	100.0%	0.893
	1–4 months	0	0.0%	14	100.0%	14	100.0%	
	5–6 months	0	0.0%	3	100.0%	3	100.0%	
Final prosthesis retention	Cemented	1	6.7%	14	93.3%	15	100.0%	0.348
	Overdenture	0	0.0%	12	100.0%	12	100.0%	
	Screwed	0	0.0%	19	100.0%	19	100.0%	
Any prosthesis repair?	Yes	1	50.0%	1	50.0%	2	100.0%	0.040*
	No	0	0.0%	48	100.0%	48	100.0%	
Peri-implant bone loss?	Yes, more than 1.5 mm	0	0.0%	2	100.0%	2	100.0%	
	No	0	0.0%	43	100.0%	43	100.0%	

*Statistically significant at p<0.05.

Regarding adverse events, four (4.7%) occurrences were reported: two (2.3%) chronic pain episodes and two (2.3%) local inflammatory reactions. No correlation was found between patient characteristics or implant-related variables and adverse events (Tables 5 and 6).

**Table 5:** Relative frequency of patient-related variables and their association with adverse events

Any adverse event occurred after surgery?		Yes		No		Total		OR (CI 95%)	p-value
		N	%	N	%	N	%		
Sex	Woman	1	1.6%	60	98.4%	61	100.0%	0.1 (0.0–1.2)	0.072
	Man	3	12.0%	22	88.0%	25	100.0%	(ref.)	
Presence of thyroid dysfunction?	Yes	0	0.0%	4	100.0%	4	100.0%	ƚ	0.824
	No	4	4.9%	78	95.1%	82	100.0%	(ref.)	
Presence of coagulation disorders?	Yes	0	0.0%	3	100.0%	3	100.0%	ƚ	0.861
	No	4	5.0%	76	95.0%	80	100.0%	(ref.)	
Presence of poor healing capacity?	Yes	0	0.0%	1	100.0%	1	100.0%	ƚ	0.952
	No	4	4.9%	78	95.1%	82	100.0%	(ref.)	
Presence of incomplete jawbone growth?	Yes	0	0.0%	5	100.0%	5	100.0%	ƚ	0.783
	No	4	4.9%	77	95.1%	81	100.0%	(ref.)	
Regular steroid use?	Yes	0	0.0%	1	100.0%	1	100.0%	ƚ	0.953
	No	4	4.8%	80	95.2%	84	100.0%	(ref.)	
Previous radiotherapy in the head/neck?	Yes	0	0.0%	1	100.0%	1	100.0%	ƚ	0.953
	No	4	4.8%	80	95.2%	84	100.0%	(ref.)	
Bisphosphonate therapy?	Yes	0	0.0%	3	100.0%	3	100.0%	ƚ	0.864
	No	4	4.9%	78	95.1%	2	100.0%	(ref.)	
Psychological limitations?	Yes	0	0.0%	17	100.0%	17	100.0%	ƚ	0.402
	No	4	5.9%	64	94.1%	68	100.0%	(ref.)	
Presence of bone metabolism disorders?	Yes	0	0.0%	4	100.0%	4	100.0%	ƚ	0.822
	No	4	4.9%	77	95.1%	81	100.0%	(ref.)	
Smoking?	Yes	0	0.0%	11	100.0%	11	100.0%	ƚ	0.568
	No	4	5.4%	70	94.6%	74	100.0%	(ref.)	

ƚ No sufficient sample size to calculate.

**Table 6:** Relative frequency of implant-related variables and their association with adverse events

Any adverse event occurred after surgery?		Yes		No		Total		OR (95% CI)	p-value
		N	%	N	%	N	%		
Implant length	10 mm	0	0.0%	17	100.0%	17	100.0%	ƚ	
	12 mm	2	5.1%	37	94.9%	39	100.0%	0.7 (0.1–5.3)	0.733
	14 mm	2	7.1%	26	92.9%	28	100.0%	(ref.)	
Region of implant placement	Lower canines	0	0.0%	4	100.0%	4	100.0%	ƚ	
	Lower central incisors	3	8.6%	32	91.4%	35	100.0%	1.7 (0.2–17.4)	0.660
	Lower molars	0	0.0%	3	100.0%	3	100.0%	ƚ	
	Lower premolars	0	0.0%	7	100.0%	7	100.0%	ƚ	
	Upper canines	0	0.0%	1	100.0%	1	100.0%	ƚ	
	Upper lateral incisors	1	5.3%	18	94.7%	19	100.0%	(ref.)	
	Upper premolars	0	0.0%	7	100.0%	7	100.0%	ƚ	
	Others	0	0.0%	10	100.0%	10	100.0%	ƚ	
Insertion torque (N.cm)	10–35	0	0.0%	22	100.0%	22	100.0%	ƚ	0.158
	36–60	4	10.3%	35	89.7%	39	100.0%	(ref.)	
Healing	Yes	3	7.0%	40	93.0%	43	100.0%	3.1 (0.3–30.8)	0.317
	No	1	2.4%	41	97.6%	42	100.0%	(ref.)	
Prosthesis type	Multi-unit fixed prosthesis	2	4.8%	40	95.2%	42	100.0%	0.6 (0.1–4.4)	0.591
	Overdenture	0	0.0%	13	100.0%	13	100.0%	ƚ	
	Single-unit prosthesis	2	8.0%	23	92.0%	25	100.0%	(ref.)	
Final prosthesis retention	Cemented	1	6.7%	14	93.3%	15	100.0%	(ref.)	0.348
	Overdenture	0	0.0%	12	100.0%	12	100.0%	ƚ	
	Screwed	0	0.0%	19	100.0%	19	100.0%	ƚ	
Any prosthesis repair was reported?	Yes	1	50.0%	1	50.0%	2	100.0%	(ref.)	0.055
	No	0	0.0%	48	100.0%	48	100.0%	ƚ	
Periimplant bone loss?	Yes, more than 1.5 mm	1	16.7%	5	83.3%	6	100.0%	(ref.)	
	No	0	0.0%	56	100.0%	56	100.0%	ƚ	

ƚ No sufficient sample size to calculate.

**Figure 1:**
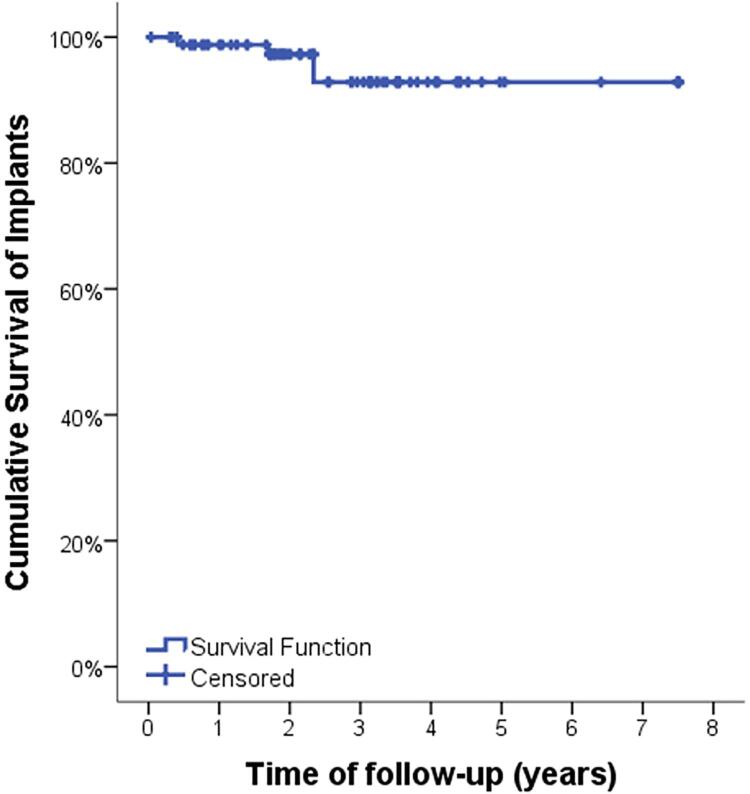
Kaplan-Meier implant survival curve

**Figure 2:**
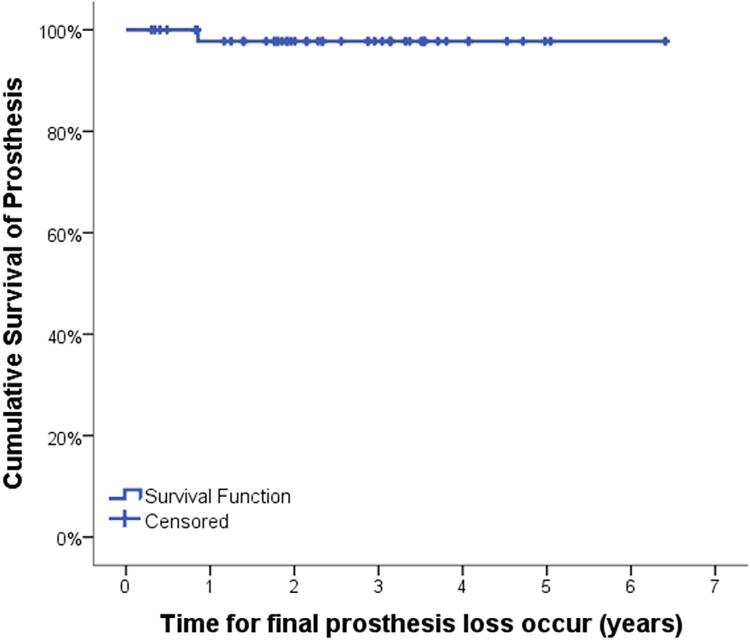
Kaplan-Meier prosthesis survival curve

**Figure 3:**
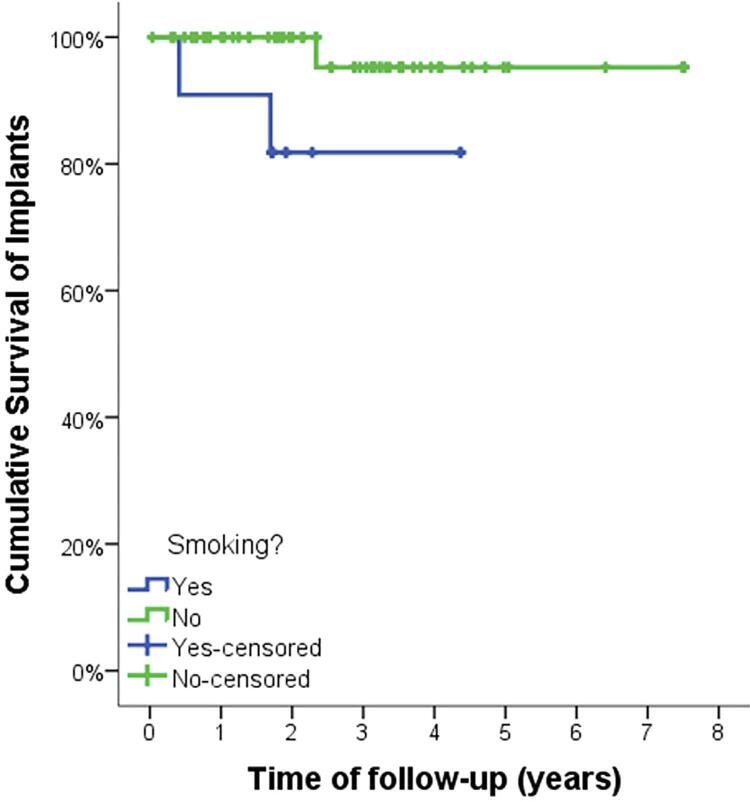
Kaplan-Meier implant survival curve according to smoking habits

Figure 1 and Figure 2 show the implants and prostheses survival analysis by Kaplan-Meier survival curves. The mean implant and prosthesis survival time was, respectively, 7.1 years and 6.3 years (Table 7). From variables that showed significance by the chi-square test, factors that could influence survival were evaluated. Thus, it was found that the mean implant survival time for non-smokers was greater than for smokers (3.7 years for smokers and 7.2 for non-smokers; p=0.002) (Figure 3 and Table 7). The mean implant and prosthesis loss time was, respectively, 1.7 years (SD: 1.9) and 0.9 years.

**Table 7:** Mean, standard deviation, and 95% confidence interval of implant and prosthesis survival, based on Kaplan-Meier survival curves

	Mean survival rate (years)	Standard deviation	95% inferior confidence interval	95% superior confidence interval	p-valor between factors
All implants	7.098	0.197	6.713	7.484	-
All prostheses	6.282	0.125	6.037	6.527	-
Implants in smokers	3.765	0.394	2.99	4.537	0.002*
Implants in non-smokers	7.255	0.17	6.92	7.588	-

*Statistically significant at p<0.05.

## Discussion

Implant-supported prostheses proved to be a good choice for the treatment of totally or partially edentulous patients. In this study, high implant (95.3%) and prosthesis (98.2%) survival rates were found in a follow-up period of up to eight years, showing that extra-narrow implants are also a reliable option, especially to rehabilitate regions with limited space. Moreover, the treatment approach used in similar situations, in which conventional implants are inserted in grafted areas, has been reported to present lower survival rates (90% at five years of follow-up).^13^ Besides the association between dental implant failure and previous augmentation techniques, several complications, such as graft exposure/loss and infections, have also been reported concerning this approach.^14^

The results obtained in this study are very similar to those obtained by other authors, presenting high long-term survival rates, ranging from 94.3%^15^ to 96.8%^5^ for extra-narrow implants. Moreover, while some authors state that a reduced-diameter implant could lead to lower survival rates when compared with standard-diameter implants, studies show that they do not differ greatly in both implant and prosthesis survival and success rates.^16,17^

It is important to identify potential risk factors associated with implant and prosthesis failure and evaluate if it is possible to manage them. However, little is discussed about the use of extra-narrow implants, specifically. At the patient level, it was reported that systemic diseases, such as diabetes and osteoporosis, are associated with an increased risk of implant failure.^18^ However, it was not observed in this study, as no correlation was found between medical conditions and extra-narrow implant loss. On the other hand, smoking habits increased by eight times the chances of extra-narrow implant loss. The tobacco use by patients with dental implants is extensively discussed and, although a study reported that smoking alone could not be considered a risk factor,^19^ other authors showed higher risks of implant failure in smokers.^20,21,22^

Regarding implant-related factors, no correlation for implant loss was found. Other authors also observed no differences in narrow implant survival rates among different types of restoration and implant placement,^7^ showing that narrow implants are a reliable treatment option, even for challenging maxillary or mandibular rehabilitation. In this study, most implants were inserted in the central and lateral incisors, which are reported to reduce the risk of implant loss, due to the absence of increased occlusal forces.^4^

However, when prosthesis loss was evaluated, a significant association was found with previous need of prosthesis repair. Although similar results were found in a study showing that all lost prostheses had previously underwent laboratory repair,^23^ there was only one prosthesis lost in this study, which is not statistically representative.

No correlation was observed between adverse events and implant loss in this study, even though implant failure was associated with the occurrence of local infections by other authors.^7^ Since no association was found between implant failure and patient characteristics or adverse events, all implant losses were results of lack of osseointegration, probably related to factors inherent to surgery and individual healing process.^24^

The mean implant survival time for narrow implants in this study, according to the Kaplan-Meier survival analysis, was slightly greater than seven years, whereas another study reported approximately 4.5 years of survival.^25^ However, since this study included only patients treated with overdentures supported by narrow implants, differences in the distribution of occlusal forces must be considered and these results must be analyzed carefully.^26^ When implants placed only in smoking patients were evaluated, their mean survival time was cut in half, showing the great effect of tobacco use on dental implant treatment, as observed by other authors.^27^ Regarding prosthesis survival, since only one failed, the mean survival time was almost the same as the mean follow-up period, as observed before.^28^

Since retrospective observational studies use data that were originally collected for other purposes, not all relevant information might have been available for analysis, and this is a limitation of this study. There could be missing data due to poor registration quality or variables that were not considered to be registered in advance. In both cases, the origin of missing information can lead to information bias. Moreover, due to this study design, it may be difficult to assess the temporal relationship between data found, leading to a potential confounding bias. Information related to date of implant and prosthesis placement were collected, as well as concerning risk factors. This information was important to analyze some temporal correlations, such as time to implant or prothesis loss. Analyses of the correlation between patient characteristics and parameters of interest may also minimize confounding bias.

## Conclusion

High implant and prosthesis survival rates were found in the long term for treatment with extra-narrow implants, showing that they are a reliable option to rehabilitate regions with limited space. Moreover, a significant correlation was observed between smoking and implant loss. Since the retrospective design of this study presented some limitations, further prospective studies must be conducted to confirm its results.

## References

[1] Adell R, Eriksson B, Lekholm U, Brånemark PI, Jemt T. Long-term follow-up study of osseointegrated implants in the treatment of totally edentulous jaws. Int J Oral Maxillofac Implants [Internet]. 1990 [cited 2022 June 23];5(4):347-59. Available from: http://www.ncbi.nlm.nih.gov/pubmed/2094653 2094653

[2] French D, Larjava H, Ofec R. Retrospective cohort study of 4591 Straumann implants in private practice setting, with up to 10-year follow-up: Part 1: multivariate survival analysis. Clin Oral Implants Res. 2015;26(11):1345-54. doi: 10.1111/clr.12463 10.1111/clr.12463 25134415

[3] Degidi M, Piattelli A, Carinci F. Clinical outcome of narrow diameter implants: a retrospective study of 510 implants. J Periodontol. 2008;79(1):49–54. doi: 10.1902/jop.2008.070248 10.1902/jop.2008.070248 18166092

[4] Yang G, Chen L, Gao Y, Liu H, Dong H, Mou Y. Risk factors and reoperative survival rate of failed narrow-diameter implants in the maxillary anterior region. Clin Implant Dent Relat Res. 2020;22(1):29-41. doi: 10.1111/cid.12867 10.1111/cid.12867 31797552

[5] King P, Maiorana C, Luthardt R, Sondell K, Øland J, Galindo-Moreno P, et al. Clinical and radiographic evaluation of a small-diameter dental implant used for the restoration of patients with permanent tooth agenesis (hypodontia) in the maxillary lateral incisor and mandibular incisor regions: a 36-month follow-Up. Int J Prosthodont. 2016;29(2):147-53. doi: 10.11607/ijp.4444 10.11607/ijp.4444 26929953

[6] Schiegnitz E, Al-Nawas B. Narrow-diameter implants: a systematic review and meta-analysis. Clin Oral Implants Res. 2018;29 Suppl 16:21-40 10.1111/clr.13272 30328192

[7] Shi JY, Xu FY, Zhuang LF, Gu YX, Qiao SC, Lai HC. Long-term outcomes of narrow diameter implants in posterior jaws: a retrospective study with at least 8-year follow-up. Clin Oral Implants Res. 2018;29(1):76-81. doi: 10.1111/clr.13046 10.1111/clr.13046 28845539

[8] Alshiddi IF, Alsahhaf A, Alshagroud RS, Al-Aali KA, Vohra F, Abduljabbar T. Clinical, radiographic, and restorative peri-implant measurements of narrow and standard diameter implants in obese and nonobese patients: a 3-year retrospective follow-up study. Clin Implant Dent Relat Res. 2019;21(4):656-61. doi: 10.1111/cid.12798 10.1111/cid.12798 31172671

[9] Andersen E, Saxegaard E, Knutsen BM, Haanaes HR. A prospective clinical study evaluating the safety and effectiveness of narrow-diameter threaded implants in the anterior region of the maxilla. Int J Oral Maxillofac Implants. 2001;16(2):217-24. 11324210

[10] Vigolo P, Givani A, Majzoub Z, Cordioli G. Clinical evaluation of small-diameter implants in single-tooth and multiple-implant restorations: a 7-year retrospective study. Int J Oral Maxillofac Implants. 2004;19(5):703-9. 15508986

[11] Buser D, Martin W, Belser UC. Optimizing esthetics for implant restorations in the anterior maxilla: anatomic and surgical considerations. Int J Oral Maxillofac Implants. 2004;19 Suppl:43-61. 15635945

[12] Wilson JP, Johnson TM. Frequency of adequate mesiodistal space and faciolingual alveolar width for implant placement at anterior tooth positions. J Am Dent Assoc. 2019;150(9):779-87. doi: 10.1016/j.adaj.2019.05.003 10.1016/j.adaj.2019.05.003 31439205

[13] Tran D, Gay I, Diaz-Rodriguez J, Parthasarathy K, Weltman R, Friedman L. Survival of dental implants placed in grafted and nongrafted bone: a retrospective study in a university setting. Int J Oral Maxillofac Implants. 2016;31(2):310-7. doi: 10.11607/jomi.4681 10.11607/jomi.4681 27004278

[14] Rabelo GD, Paula PM, Rocha FS, Jordão Silva C, Zanetta-Barbosa D. Retrospective study of bone grafting procedures before implant placement. Implant Dent. 2010;19(4):342-50. doi: 10.1097/ID.0b013e3181e416f9 10.1097/ID.0b013e3181e416f9 20683291

[15] Pommer B, Mailath-Pokorny G, Haas R, Buseniechner D, Millesi W, Fürhauser R. Extra-short (< 7 mm) and extra-narrow diameter (< 3.5 mm) implants: a meta-analytic literature review. Eur J Oral Implantol. 2018;11 Suppl 1:S137-46. 30109305

[16] Ma M, Qi M, Zhang D, Liu H. The clinical performance of narrow diameter implants versus regular diameter implants: a meta-analysis. J Oral Implantol. 2019;45(6):503-8. doi: 10.1563/aaid-joi-D-19-00025 10.1563/aaid-joi-D-19-00025 31536434

[17] Souza AB, Sukekava F, Tolentino L, César-Neto JB, Garcez-Filho J, Araújo MG. Narrow- and regular-diameter implants in the posterior region of the jaws to support single crowns: a 3-year split-mouth randomized clinical trial. Clin Oral Implants Res. 2018;29(1):100-7. doi: 10.1111/clr.13076. Epub 2017 10.1111/clr.13076 28994192

[18] Aghaloo T, Pi-Anfruns J, Moshaverinia A, Sim D, Grogan T, Hadaya D. The effects of systemic diseases and medications on implant osseointegration: a systematic review. Int J Oral Maxillofac Implants. 2019 Suppl;34:s35-s49. doi: 10.11607/jomi.19suppl.g3 10.11607/jomi.19suppl.g3 31116832

[19] Sverzut AT, Stabile GA, Moraes M, Mazzonetto R, Moreira RW. The influence of tobacco on early dental implant failure. J Oral Maxillofac Surg. 2008;66(5):1004-9. doi: 10.1016/j.joms.2008.01.032 10.1016/j.joms.2008.01.032 18423293

[20] Chrcanovic BR, Albrektsson T, Wennerberg A. Smoking and dental implants: a systematic review and meta-analysis. J Dent. 2015;43(5):487-98. doi: 10.1016/j.jdent.2015.03.003 10.1016/j.jdent.2015.03.003 25778741

[21] Naseri R, Yaghini J, Feizi A. Levels of smoking and dental implants failure: a systematic review and meta-analysis. J Clin Periodontol. 2020;47(4):518-28. doi: 10.1111/jcpe.13257 10.1111/jcpe.13257 31955453

[22] Moraschini V, Barboza EP. Success of dental implants in smokers and non-smokers: a systematic review and meta-analysis. Int J Oral Maxillofac Surg. 2016;45(2):205-15. doi: 10.1016/j.ijom.2015.08.996 10.1016/j.ijom.2015.08.996 26385308

[23] Padovan LE, Suzuki D, Kluppel LE, Vianna CP, Caldas W, Trojan LC. Factors influencing implant and prosthesis survival in zygomatic implant-supported fixed rehabilitation: a retrospective study. Odontology. 2021;109(4):965-72. doi: 10.1007/s10266-021-00621-4 10.1007/s10266-021-00621-4 34146176

[24] Esposito M, Hirsch JM, Lekholm U, Thomsen P. Biological factors contributing three major determinants for late implant failures in the Brånemark system. Eur J Oral Sci. 1998;106(1):527-51. doi: 10.1046/j.0909-8836..t01-2-.x 10.1046/j.0909-8836..t01-2-.x 9527353

[25] Rujiraphan T, Suphangul S, Amornsettachai P, Thiradilok S, Panyayong W. Clinical outcomes of small-diameter implant-retained overdentures : a retrospective analysis. J Osseointegration. 2021;13(4):191-7. doi: 10.23805/JO.2021.13.04.5

[26] Miyaura K, Morita M, Matsuka Y, Yamashita A, Watanabe T. Rehabilitation of biting abilities in patients with different types of dental prostheses. J Oral Rehabil. 2008;27(12):1073-6. doi: 10.1046/j.1365-2842.2000.00620.x 10.1046/j.1365-2842.2000.00620.x 11251780

[27] Koldsland OC, Scheie AA, Aass AM. Prevalence of implant loss and the influence of associated factors. J Periodontol. 2009;80(7):1069-75. doi: 10.1902/jop.2009.080594 10.1902/jop.2009.080594 19563286

[28] Corbella S, Alberti A, Calciolari E, Francetti L. Medium- and long-term survival rates of implant-supported single and partial restorations at a maximum follow-up of 12 years: a retrospective study. Int J Prosthodont. 2021;34(2):183-91. doi: 10.11607/ijp.6883 10.11607/ijp.6883 33882565

